# Do Small Canopy Gaps Created by Japanese Black Bears Facilitate Fruiting of Fleshy-Fruited Plants?

**DOI:** 10.1371/journal.pone.0130956

**Published:** 2015-07-24

**Authors:** Kazuaki Takahashi, Kaori Takahashi, Izumi Washitani

**Affiliations:** 1 Faculty of Tourism and Environmental Studies, Nagano University, Ueda, Nagano, Japan; 2 Division of Gene Research, Department of Life Science, Research Center for Human and Environmental Sciences, Shinshu University, Ueda, Nagano, Japan; 3 Biodiversity and Ecosystem Restoration, Institute of Agriculture and Life Sciences, The University of Tokyo, Bunkyo-ku, Tokyo, Japan; The Ohio State University, UNITED STATES

## Abstract

Japanese black bears often break branches when climbing trees and feeding on fruit in canopies, thereby creating small canopy gaps. However, the role of black bear-created canopy gaps has not been evaluated in the context of multiple forest dynamics. Our hypothesis was that small canopy gaps created by black bears improve light conditions, which facilitates fruiting of adult fleshy-fruited plants located beneath the gaps, and also that this chain interaction depends on interactions among the size of gaps, improved light conditions, forest layers, and life form of plants. The rPPFD, size of black bear-created canopy gaps, and fruiting/non-fruiting of fleshy-fruited plants were investigated in five forest layers beneath black-bear-created canopy gaps and closed canopies of Mongolian oak (*Quercus crispula*). We found that light conditions improved beneath black bear-disturbed trees with canopy gaps of large size, and the effect of improvement of light conditions was reduced with descending forest layers. Fruiting of fleshy-fruited plants, especially woody lianas and trees, was facilitated by the improvement of light conditions accompanied by an increase in the size of black-bear-created gaps. Data from this study revealed that canopy disturbance by black bears was key for improving light conditions and accelerating fruiting of fleshy-fruited trees and woody lianas in the canopy layers in particular. Therefore, our hypothesis was mostly supported. Our results provide evidence that Japanese black bears have high potential as ecosystem engineers that increase the availability of resources (light and fruit in this study) to other species by causing physical state changes in biotic materials (branches of *Q*. *crispula* in this study).

## Introduction

**Fig 1:**
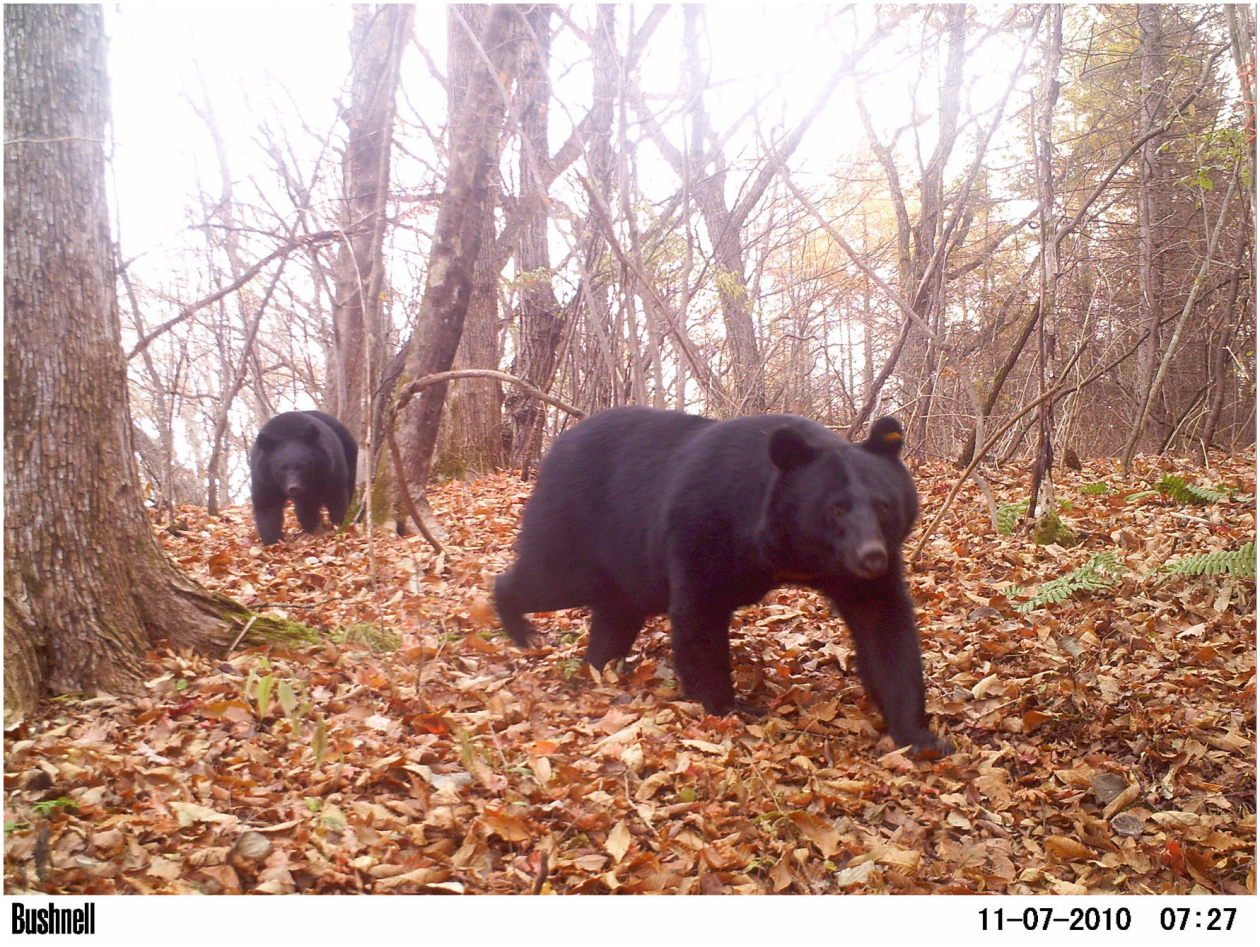
Japanese black bears (*Ursus thibetanus japonicus*). The species is distributed throughout broadleaved deciduous forests in central Japan.

**Fig 2:**
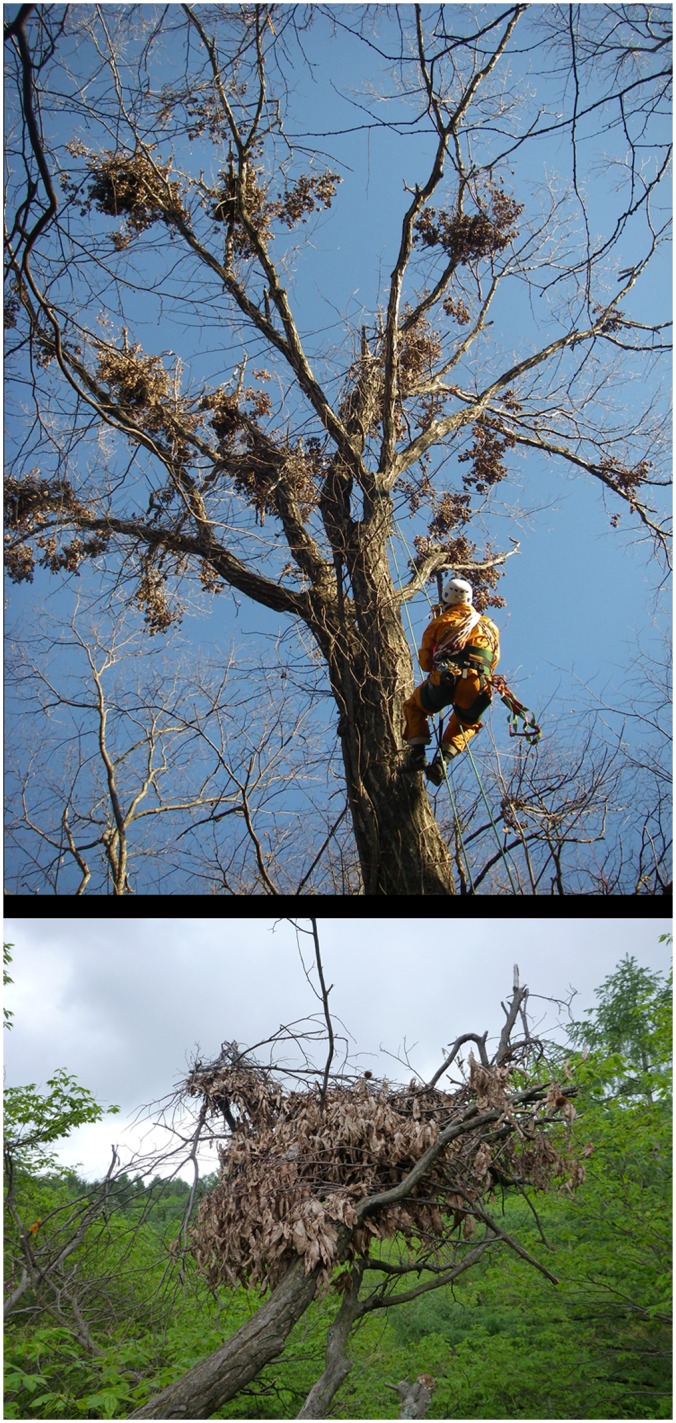
Bear shelves known as ‘Kuma-dana’ in Japanese. These shelves are made of branches of Japanese white oak (*Quercus serrata*) (above) and Japanese Chestnut (*Castanea crenata*) (below) that are broken by Japanese black bears (*Ursus thibetanus japonicus*).

**Fig 3:**
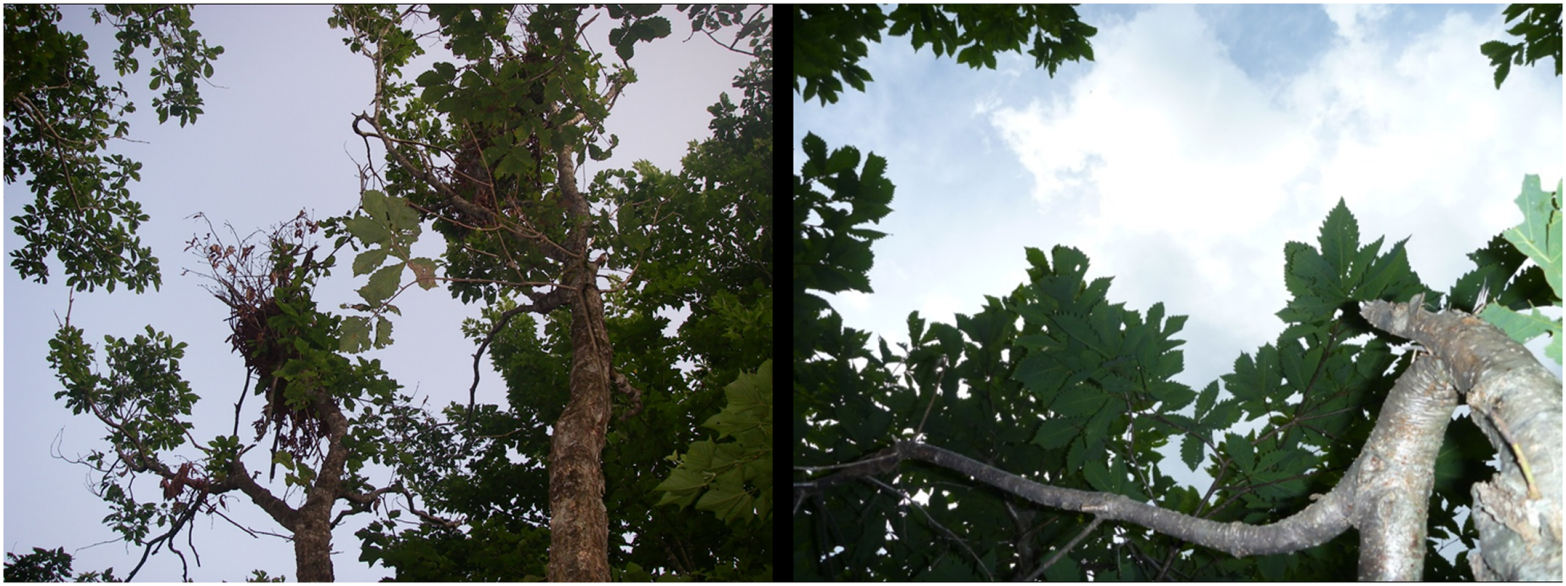
Small canopy gaps created by black bears in Mongolian oak (*Quercus crispula*) canopies.

The Japanese black bear (*Ursus thibetanus japonicus* Schlegel; Fig 1), a large-bodied omnivore, often eats fruit in the forest canopy, as well as fruit that has fallen to the forest floor. To access canopy fruit, black bears climb trees and often break branches bearing fruit. Because they frequently place broken branches across the forks of intact branches, broken branches placed in this manner are often used as spoor when investigating the feeding habits and estimating the population sizes of black bears in Japan [1, 2]. Because these broken branches look like shelves, they are known as ‘bear shelves’ [1], or ‘Kuma-dana’ in Japanese (Fig 2). Black bears feed on various fruits, including approximately 10 nut/acorn-producing tree species, such as the Japanese beech (*Fagus crenata* Blume and *F*. *japonica* Maxim), Mongolian oak (*Quercus crispula* Blume), and Japanese white oak (*Q*. *serrata* Murray), and approximately 70 fleshy-fruited plant species, such as members of the Rose (*Rosaceae*), Mulberry (*Moraceae*), Dogwood (*Cornaceae*), Grape (*Vitaceae*), and Chinese gooseberry (*Actinidiaceae*) families [3], and often create bear shelves in *Prunus* trees in early summer, and in *Quercus* and *Fagus* trees in autumn in Japan. When many branches are broken, openings without leaves or branches, small partial canopy gaps are created in the canopy (Fig 3).

In general, openings in the canopy that are caused by the fall of one or multiple trees, or tree-fall gaps, are referred to as canopy gaps [4]. The size of tree-fall gaps ranges from 10 to >1000 m^2^ in forests throughout the world [5]. Previous studies on gap dynamics examined the role that canopy gaps play in promoting the replacement and coexistence of competing tree species, and therefore in maintaining tree diversity [6–8]. The formation of canopy gaps creates heterogeneity in the abiotic environment and increases the availability of soil moisture and nutrients [9], particularly the amount of light passing through forest layers below the canopy [10]. The gap disturbance regime promotes the germination of seeds buried in soil [11] and the growth of seedlings and saplings [12]. It also provides a wide range of regeneration sites for tree species with different life forms and environmental requirements [4]. These studies have clearly contributed to our understanding of the responses of seeds, seedlings, and saplings of different tree species to the formation of canopy gaps. Similarly, many studies have emphasized responses by adult shrubs, woody lianas, and trees beneath canopy gaps after gap formation and have revealed that adults can increase their growth and reproduction, such as flowering and fruiting, because of increased light availability through canopy openings [13–15]. On the other hand, few studies have emphasized the responses of plants to branch-fall gaps < 10 m^2^ [16]. Canopy gaps formed by branch-falls are generally smaller than those formed by standing snags, trunk breaking, or uprooting [17]. However, even if such small branch-fall gaps are formed, the improvement in light conditions caused by the formation of small gaps would affect growth and reproduction of plants immediately beneath the gaps, particularly adult small trees and woody lianas in the sub-canopy layers, within a few years of gap formation. However, this idea has not been thoroughly examined.

Our previous research found that the average total size of black-bear-created gaps in a canopy is 6.9 m^2^ (minimum and maximum: 0.7 and 36.2 m^2^, respectively), considerably smaller than that of common tree-fall gaps, and also that the annual rate of bear-created canopy gap formation reaches 141.3 m^2^ ha^–1^ yr^–1^ and is approximately 6.6-fold that of tree-fall gap formation on ridges, which were hot spots of black bear activity [18]. Although we did not quantify the frequency of branch breaking by black bears, it is clearly higher than that caused by wind or other factors. Therefore, black-bear-created canopy gaps, as well as small gaps created by tree falls, have the potential to alter abiotic and biotic environments in forests. However, very little is known about the role played by canopy disturbances created by black bears in relation to light conditions and plant habitats.

Our general hypothesis was that small canopy gaps created by black bears improve light conditions and therefore facilitate fruiting by plants beneath the gaps, and also that the particulars of this sequence depend on interactions among gap size, improved light conditions, forest layers, and plant life forms. We focused on fleshy-fruited plants because they dominate mid-successional plant communities in many regions [19] and are important food resources for black bears. Our goals were to test (1) whether light conditions and the fruiting/non-fruiting of fleshy-fruited plants differ between locations below black-bear-created canopy gaps and closed canopies; (2) whether light conditions beneath canopy trees are affected by the size of black-bear-created canopy gaps and forest layers; and (3) whether the fruiting/non-fruiting of fleshy-fruited plants is influenced by differences in the gap size, light conditions, forest layers, and life forms of the plants. If this hypothesis is supported, it would provide evidence that black bears act as ‘ecosystem engineers’ [20] that physically change the abiotic environment, thereby affecting the local biota. This would add a new perspective to the study of gap dynamics that involves understanding how multiple biological interactions affect forest dynamics. Based on our results, we discuss direct and indirect interactions between black bears, canopy trees, and fleshy-fruited plants, and the role of the Japanese black bear as an ecosystem engineer.

## Materials and Methods

### Ethics Statement

This study was approved by permit from the Ministry of Environment, Japan. An ethics statement is not required for this work. However, all work was carried out within the guidelines of the Mammal Society of Japan and the Wildlife Research Center of Kyoto University.

### Study site

This study was conducted in a broad-leaved deciduous forest in Nagakura-yama national park, Karuizawa city, near Mt. Asama (2568 m), Nagano Prefecture, central Japan (36°23’N, 138°36’–37’E; 1120–1160 m a.s.l.). The annual mean temperature was 8.6°C (minimum and maximum: –15.9 and 32.3°C, respectively) and the annual rainfall was 1310.0 mm. The average maximum snow depth in winter was approximately 21.3 cm. These weather data were calculated from data recorded between 2006 and 2008 at Karuizawa Local Meteorological Observatory, about 12 km from the study site. To understand the composition of dominant trees, we measured diameter at breast height (DBH) for canopy trees > 15-cm DBH in 2008 in eight 20 × 50-m plots at the study site. The deciduous forests were dominated by Mongolian oak (*Q*. *crispula*), Japanese chestnut (*Castanea crenata* Siebold et Zucc.), and Japanese white oak (*Q*. *serrata*), which accounted for 25.8%, 20.2%, and 3.9% of the total basal area of the canopy trees, respectively. The other canopy tree species consisted of fleshy-fruited species such as *Kalopanax septemlobus* (Thunb.) Koidz. (2.9%), *Cornus controversa* Hemsl. ex Prain (1.9%), *Cerasus jamasakura* (Siebold ex Koidz.) H.Ohba (1.7%), *Padus grayana* (Maxim.) C.K.Schneid. (1.1%), *Ilex macropoda* Miq. (0.7%), *Chengiopanax sciadophylloides* (Franch. et Sav.) C.B.Shang et J.Y.Huang (0.9%), *Cerasus leveilleana* (Koehne) H.Ohba (0.6%), and *Magnolia obovata* Thunb. (0.5%), and wind-dispersed species such as *Ulmus davidiana* Planch. var. *japonica* (Rehder) Nakai (8.0%), *Pinus densiflora* Siebold et Zucc. (7.9%), *Alnus hirsuta* (Spach) Turcz. ex Rupr. var. *hirsuta* (4.4%), *Acer pictum* Thunb. (3.1%), *Acer amoenum* Carrière (2.0%), and *Acer sieboldianum* Miq. (0.7%). Japanese black bears are not considered threatened in the study region, and the area included the core ranges of several populations in the Honshu region. The population size in this area was approximately 3.3–3.9 individuals/km^2^ [21].

### Study canopy tree selection

Because Japanese black bears preferentially feed on Fagaceae nuts and acorns in autumn before hibernation [22], particularly those of Mongolian Oak (*Q*. *crispula*), which is their most important food in central Japan [23], we focused on adult *Q*. *crispula* trees. *Quercus crispula* is a deciduous oak that is widely distributed in Japan; it is one of the main constituents of secondary and natural forests. It is a monoecious tree whose wind-pollinated flowers bloom in spring as soon as the shoots elongate, and its acorns mature in the following autumn. In the study area, a good mast year for *Q*. *crispula* occurred in 2006. Black bears climbed *Q*. *crispula* trees and created ~26 bear shelves/ha during autumn 2006. We examined a core area of *Q*. *crispula* trees with bear shelves within our study area (~112 km^2^) [18]. Eight adjacent 20 × 50-m plots were placed along gentle mountain ridges (slope angle < 15°) in the core area between November 2006 and May 2007 and, as much as possible, the locations of our study plots were selected based on the uniformity of canopy tree and fleshy-fruited plant species composition, trees heights and DBHs, the previous year’s black-bear disturbance, and topography. Seventeen *Q*. *crispula* trees with bear shelves and seventeen without shelves were randomly selected in the study plots in June 2007. There was no significant difference in tree height or DBH between black-bear-disturbed (average height ± SD = 16.5 ± 3.4 m; average DBH ± SD = 35.6 ± 10.9 cm) and non-disturbed trees (average height ± SD = 17.5 ± 3.4 m; average DBH ± SD = 34.2 ± 8.9 cm) (t-test: *p* > 0.05).

### Light availability and gap definition

Light conditions beneath the study trees were assessed by measuring relative photosynthetic photon flux density (rPPFD). Hemispherical photographs were taken at heights of 0.5, 2, and 5 m above the forest floor, and at both bottom canopy positions (average height ± SD = 7.6 ±1.3 m) and the highest positions we could reach by climbing (average height ± SD = 9.4 ± 1.4 m) just beneath the canopy center of a study tree during a foliated period (July—August 2007). We used climbing tools, such as harnesses, climbing ropes, ascenders, and descenders, to reach each point, and a Nikon Coolpix 5400 digital camera with a Nikkor 7 mm FC-E9 fisheye lens and Nikon UR-E10 fisheye converter adapter to obtain photographs. All measurements were taken when the sky was nearly overcast or either 1 h after dawn or 1 h before dusk. The rPPFDs were calculated using a total of 510 photographs and a Gap Light Analyzer v2.0 [24]; the calculation involved averaging three photographs from each point.

The middle and top canopy layer of each study tree was defined as intermediate between the bottom and top of the study tree’s canopy, and as within one vertical meter of the top of the study tree’s canopy, respectively. Because we could not obtain rPPFD data from the middle and top canopy layers, the rPPFDs of these layers were estimated using the following exponential function:

$$\text{rPPFD}=a\cdot e^{b\;\cdot {\text{d}}_{\text{canopy layer}}}$$ (1)

where d_canopy layer_ is the vertical distance downward from the top of a study tree’s canopy to the middle or top canopy layer of the study tree, and *a* and *b* are coefficients. Because light intensity in a forest exponentially decreases with increasing leaf area index at greater vertical distances from the canopy in accordance with Beer’s law [25, 26], we used an exponential function whose independent variable is the vertical distance from the top of each study tree’s canopy. To estimate the rPPFDs of the middle and top canopy layers of each study tree, we used height data from four observation points (0.5, 2, and 5 m above the forest floor, and the bottom canopy layer) converted to vertical distance from the top of each tree’s canopy, and then determined coefficients *a* and *b* by substituting data for rPPFDs and vertical distances obtained from each study tree.

**Fig 4:**
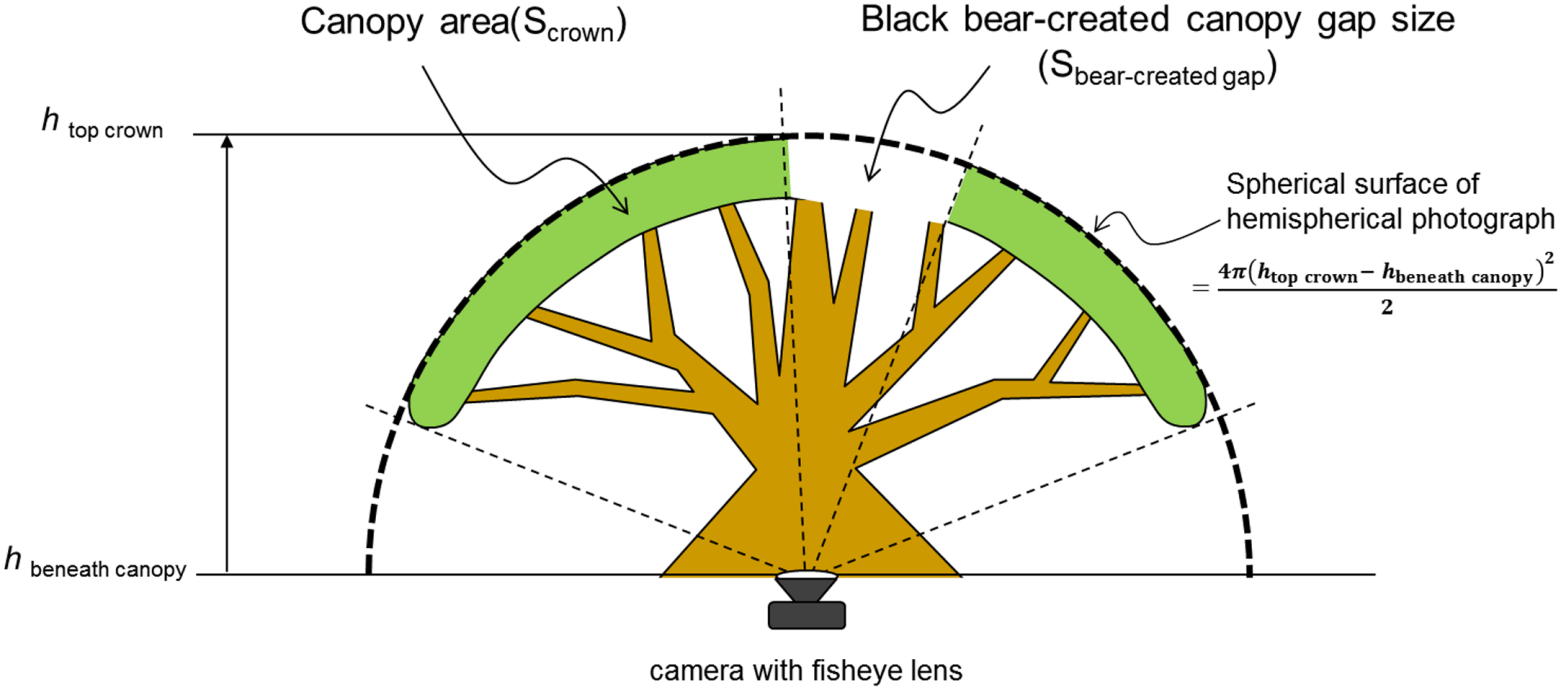
Estimation of Japanese black-bear-created canopy gap size using hemispherical photographs and Gap Light Analyzer (v2.0) software (Frazer, Canham & Lertzman 1999).

The size of black-bear-created canopy gaps was estimated using the hemispherical photographs used in assessing rPPFDs above (Fig 4) [18].

The size of each black-bear-created canopy gap was calculated using the following function:

$$\begin{array}{l} {\text{S}}_{\text{bear}-\text{created gap}}={\text{ S}}_{\text{crown }}\times {\text{ Sp}}_{\text{ \% bear}-\text{created gap}/\text{crown }}\\ \;\;\;\;\;\;\;\;\;\;\;\;\;\;\;\;\;\;=\left\{{\text{ Sp}}_{\text{\% crown }}\times \;\frac{4\pi {\left(h_{\text{top crown}}-\;h_{\text{beneath canopy}}\right)}^{2}}{2}\right\}\;\times {\text{ Sp}}_{\text{ \% bear}-\text{created gap}/\text{crown }} \end{array}$$ (2)

where S_bear-created gap_ (m^2^) is the black-bear-created canopy gap size, S_crown_ (m^2^) is the crown size, Sp_% bear-created gap/crown_ (%) is the percentage of bear-created canopy gap area in relation to the total area of the crown, Sp_% crown_ (%) is the percentage of the crown area in relation to the total area of a hemispherical photograph, *h*_top crown_ (m) is the tree height, and *h*_beneath canopy_ (m) is the height of a position immediately below the crown of a study tree.

### Fruiting investigation

Abundance of ripe fleshy-fruits varies among years and fluctuates asynchronously among fruiting species [27]. The effect of light condition on the fruit production of plants in tree-fall gaps also differs among species [15]. However, in general, in temperate regions, most perennial plants form flower buds for the current summer during the previous season [28]. That is, flowers develop using photosynthetic products gained in the previous season and fruits develop and ripen using resources gained during the current season [29]. Therefore, we assumed that differences among plant species in their responses to improved light conditions are small, and also considered that changes in light conditions beneath *Q*. *crispula* trees, because of the creation of bear shelves in autumn 2006, affected the development of flower buds on many plant species beneath the trees in 2007 and also the development and ripening of fruits in 2008. Incidentally, the fruits of many autumn-fruiting species of fleshy-fruited plants ripened in the study site in 2008.

**Fig 5:**
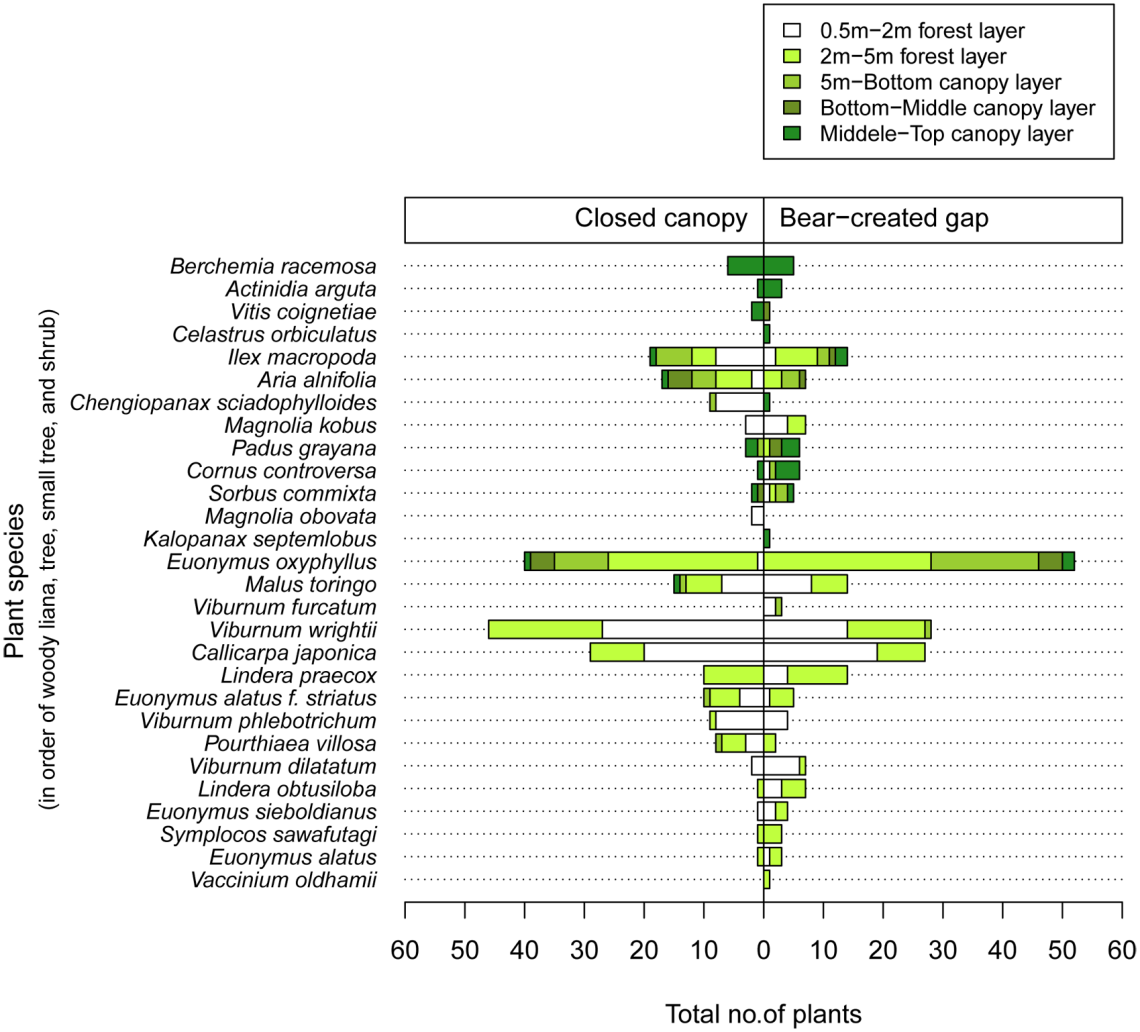
Species composition of fleshy-fruited plants in different forest layers beneath Japanese black-bear-created canopy gaps and closed canopies of Mongolian oak (*Quercus crispula*).

Thus, in autumn 2008, which is the fruiting season of most fleshy-fruited plants in central Japan [30], we investigated the presence or absence of fleshy-fruits on shrubs, woody lianas, and trees that occurred within a 5-m radius from the center of a study tree’s canopy, in five forest layers (0.5–2 m, 2–5 m, 5 m—bottom canopy, bottom canopy—middle canopy, and middle canopy—top canopy) using climbing tools and binoculars. In total, 618 fleshy-fruited plants of 28 species—including woody lianas, trees, small trees, and shrubs—were recorded beneath both black-bear-created gaps and closed canopies (Fig 5). We found significant positive correlations of species rank order in the numbers of conspecific fleshy-fruited plants between black bear-disturbed and non-disturbed trees (Spearman’s rank correlation test, *σ* = 0.64, *P* < 0.001). This indicates that the quantitative compositions of fleshy-fruited plants are similar between black bear-disturbed and non-disturbed trees.

In the middle latitudes of the northern hemisphere, direct sunlight that passes through normally sized tree-fall gaps is usually not focused on the forest floor directly below the canopy gap because of the low altitude of the sun in early summer [10]. In the case where black-bear-created gaps are smaller than common tree-fall gaps, we observed that direct sunlight through the canopy openings is often intercepted by canopies of small trees just beneath the gaps, resulting in only diffuse light penetrating to the understory, with no northern deviation. Thus, we considered that there is little position discrepancy between where diffuse light reaches and where fruiting investigations occurred in each forest layer.

### Statistical analyses

We used generalized linear models (GLMs) with a Gaussian distribution and identity link function to determine the effects of canopy disturbances created by black bears, black-bear-created gap sizes, and forest layers on rPPFDs beneath the 34 *Q*. *crispula* trees. To account for the fruiting or non-fruiting of all 618 fleshy-fruited plants that occurred beneath the 34 target *Q*. *crispula* trees, we used generalized linear mixed models (GLMMs) with a Binomial distribution and log link function. We used canopy disturbances by Japanese black bears, size of black-bear-created gaps, rPPFDs, forest layers, the life form of any fleshy-fruited plants, the interaction between canopy disturbance by black bears and rPPFDs, the interaction between gap size and rPPFDs, and the interaction between forest layers and rPPFDs as explanatory variables, and the species of any fleshy-fruited plants as a random effect, using individual data on all 618 fleshy-fruited plants that occurred near black-bear-disturbed and non-disturbed trees (Fig 4). Because life form is a categorical variable, we used woody lianas as a reference life form category in the GLMMs. In addition to any positive effect of canopy disturbance by black bears or the size of black-bear-created gaps, we considered that our hypothesis was supported if we observed either or both of the following effects: (1) a positive effect of the interaction between canopy disturbance by black bears and rPPFDs on the fruiting of fleshy-fruited plants, and/or (2) a positive effect of the interaction between gap size and rPPFDs on fruiting of fleshy-fruited plants. The former indicates that improvement of light conditions due to canopy disturbance by black bears facilitates the fruiting of fleshy-fruited plants beneath the canopy, and the latter indicates that improvement in light conditions accompanied by increases in the size of black-bear-created gaps facilitates fruiting of fleshy-fruited plants beneath the canopy. We calculated the Akaike Information Criterion (AIC) for all possible combinations of explanatory variables in the GLMs and GLMMs. The model with the lowest AIC was considered superior, and explanatory variables included in the best model were considered to represent the factors that affected rPPFDs and fruiting/non-fruiting of any fleshy-fruited plants. All GLM and GLMM analyses were carried out using R version 3.0.3 [31].

## Results

### Light availability

**Fig 6:**
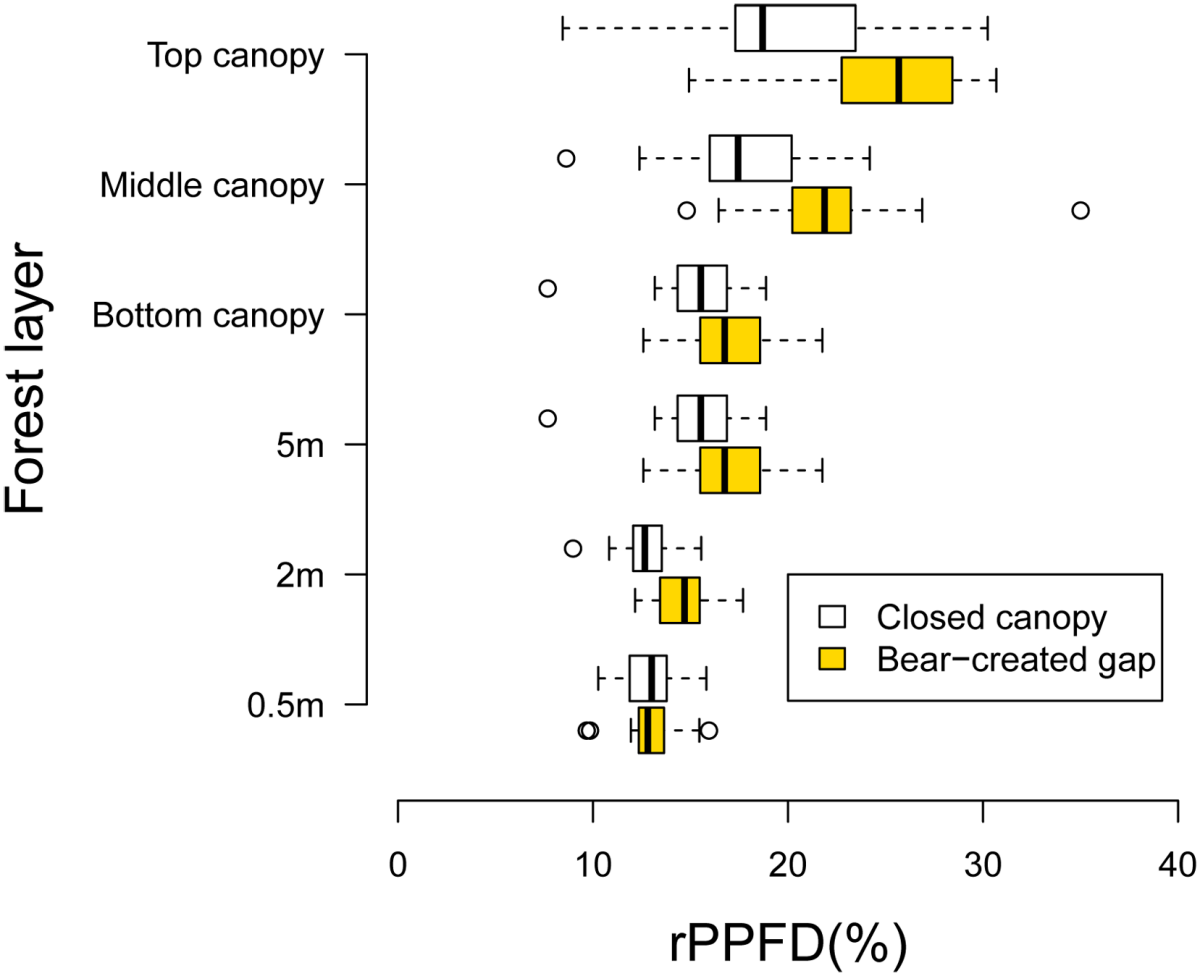
rPPFD in different forest layers beneath Japanese black-bear-created canopy gaps (solid bars) and closed canopies (open bars) of Mongolian oak (*Quercus crispula*). Each box represents an interquartile range, and the vertical line inside the box indicates the median. The whiskers extend to the lowest and highest values below and above the first and third quartile, respectively, excluding outliers. Circles represent outliers that are between 1.5 and 3.0 times the interquartile range.

The average percentages of improvement in rPPFDs beneath black-bear-created gaps vs. those beneath closed canopies were approximately 33%, 26%, 19%, 19%, 15%, and 0% in the top, middle, and bottom canopy, and the 5-m, 2-m, and 0.5-m forest layers, respectively (Fig 6). In the top canopy layers, the rPPFD beneath black-bear-created gaps and in closed canopies ranged from 14.9% to 55.5% (average = 26.4, SD = 8.5) and from 8.4% to 30.2% (average = 19.8, SD = 5.3), respectively (Fig 6).

The best GLM model showed that canopy disturbances created by black bears, gap sizes, and forest layers had significant and positive effects on rPPFD (Table 1).

**Table 1:** Results of null and best models selected from all generalized linear models (GLM) on the effects of canopy disturbances by Japanese black bears, size of black-bear-created gaps, and forest layers on relative photosynthetic photon flux density (rPPFD) beneath Mongolian oaks (*Quercus crispula*).

Model	AIC	Response variable	Explanatory variable	Coefficient	*p*
**Null**	1272.9	rPPFD	1		
**Best (Full)**	1125.2	rPPFD	Bear disturbance	2.10	**
			Gap size	0.59	***
			Forest layer	0.18	*

*:*p* < 0.05,

**:*p* < 0.01,

***:*p* < 0.001

### Fruiting of fleshy-fruited plants

**Fig 7:**
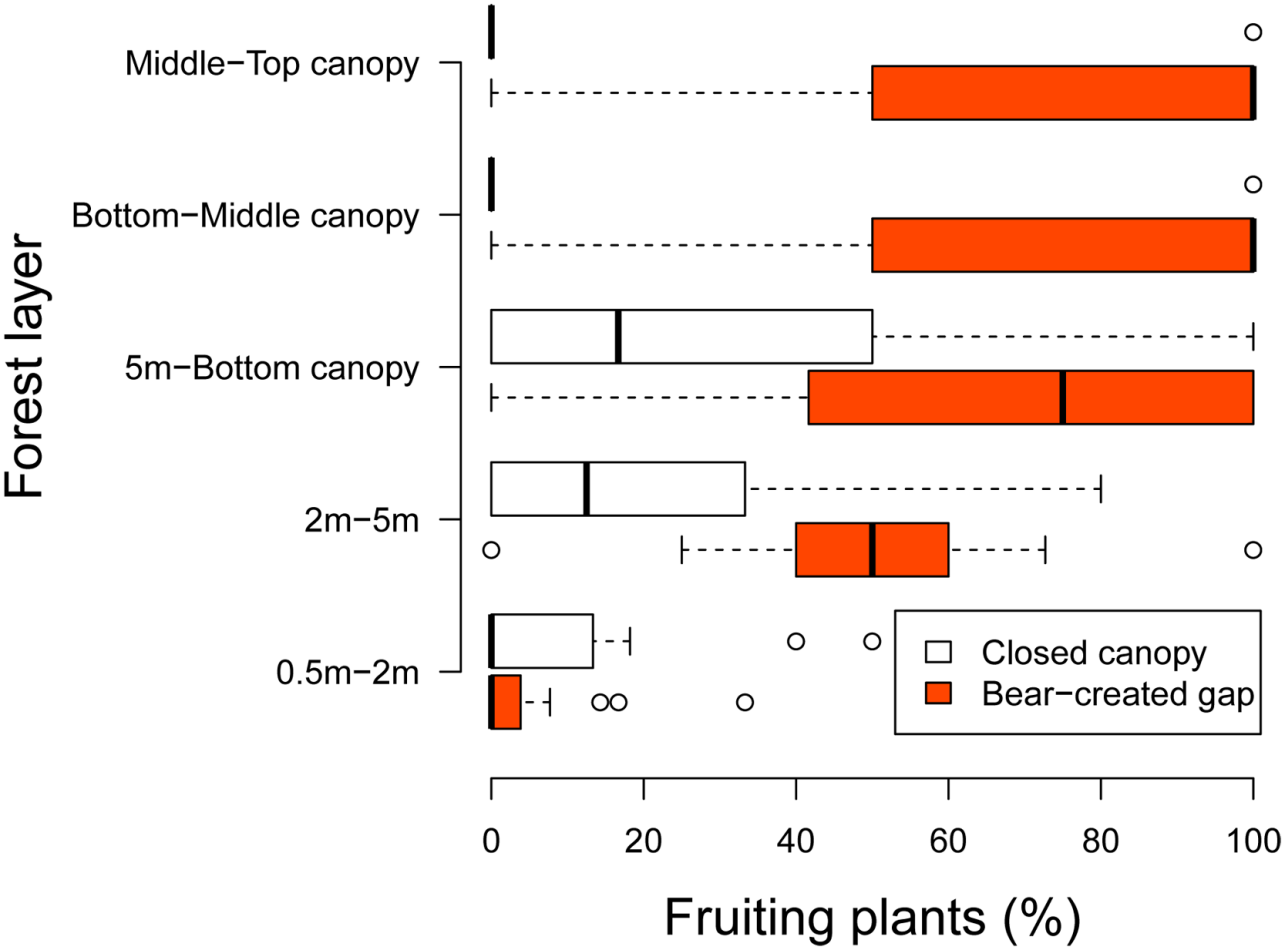
Rates of fruiting by fleshy-fruited plants (a) and plant species (b) in different forest layers beneath Japanese black-bear-created canopy gaps (solid bars) and closed canopies (open bars) of Mongolian oak (*Quercus crispula*). See Fig 6 for an explanation of the statistics indicated by the boxplots.

In black-bear-created gaps, average percentages of fruiting plants (no. of fruiting plants / no. of fruiting + non-fruiting plants) occurring in each forest layer increased: 4.5% (SD = 9.4) in the 0.5–2 m layer, 50.2% (SD = 27.3) in the 2–5 m layer, 65.9% (SD = 39.9) in the 5 m—bottom canopy layer, 75.0% (SD = 41.8) in the bottom—middle canopy layer, and 80.0% (SD = 35.0) in the middle—top canopy layer (Fig 7). For closed canopies, average percentages of fruiting plants in the 0.5–2 m, 2–5 m, 5 m—bottom canopy, bottom—middle canopy, and middle—top canopy layers were approximately 8.9% (SD = 16.0, n = 17), 18.6% (SD = 23.8, n = 17), 34.5% (SD = 41.1, n = 17), 22.2% (SD = 44.1, n = 17), and 10.0% (SD = 31.6, n = 17), respectively (Fig 7).

**Fig 8:**
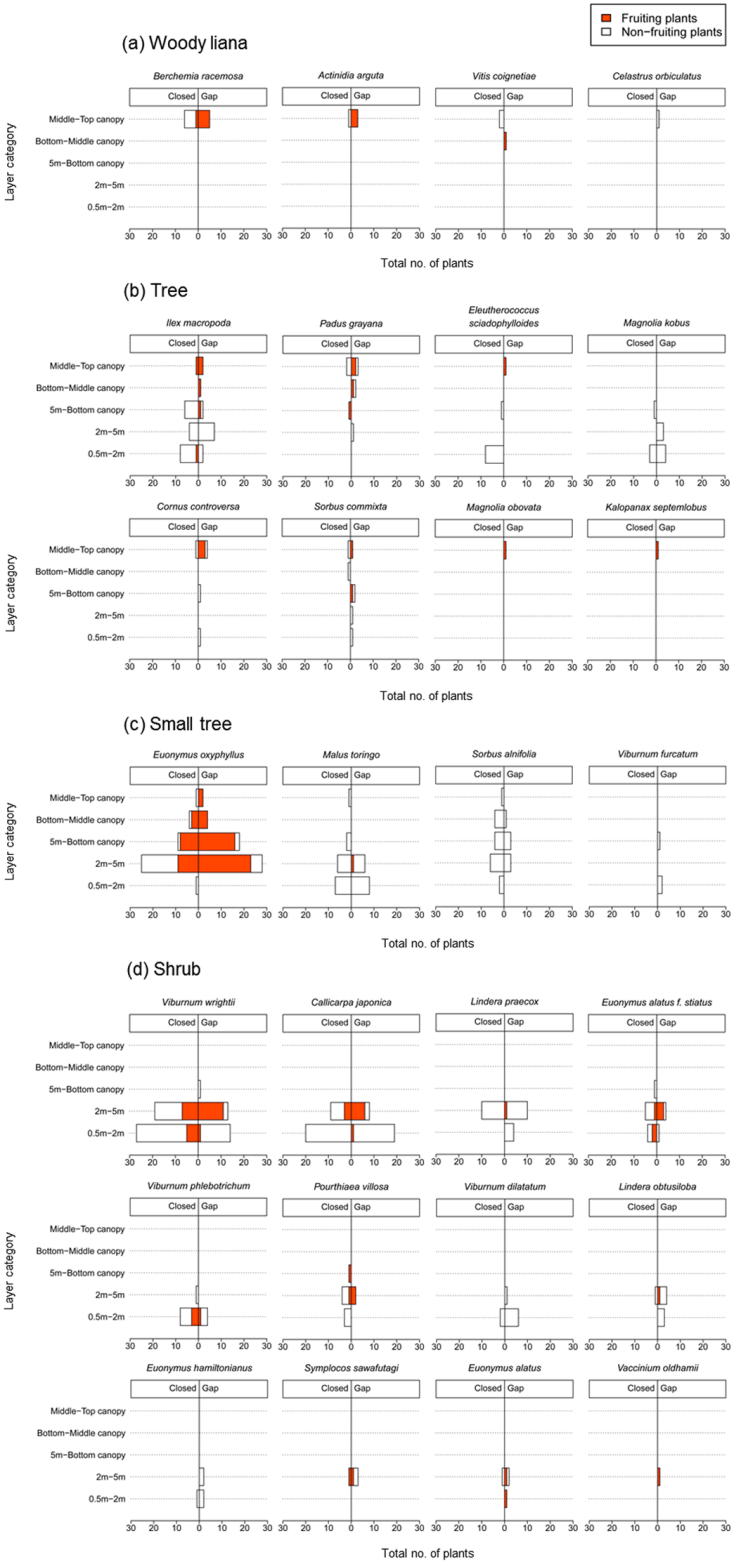
Fleshy-fruited plants with fruits and without fruits in different forest layers beneath Japanese black-bear-created canopy gaps and closed canopies of Mongolian oak (*Quercus crispula*).

Four woody liana species occurred in the bottom—middle and/or middle—top canopy layers beneath black-bear-created gaps and/or closed canopies (Fig 8a). Of these, most individuals of *Berchemia racemosa* and all individuals of *Actinidia arguta* and *Vitis coignetiae* that occurred beneath closed canopies lacked ripe fruit, but all individuals of these three species that occurred beneath black-bear-created gaps had ripe fruit (Fig 8a). Eight tree species occurred from the 0.5–2 m forest layers to the middle—top canopy layers beneath black-bear-created gaps and/or closed canopies (Fig 8b). Of these species beneath closed canopies, only *Ilex macropoda* and *Padus grayana* had individuals with ripe fruit, whereas all seven tree species that occurred beneath black-bear-created gaps had individuals with ripe fruit (Fig 8b). Four small tree species occurred from the 0.5–2 m forest layers to the middle—top canopy layers beneath black-bear-created gaps and/or closed canopies (Fig 8c). Of these small trees, although many individuals of *Euonymus oxyphyllus* had ripened fruit beneath both black-bear-created gaps and closed canopies, only *Malus toringo* individuals beneath black-bear-created gaps had ripe fruit (Fig 8c). Twelve shrub species occurred from the 0.5–2 m forest layers to the 5 m—bottom canopy layers beneath black-bear-created gaps and/or closed canopies (Fig 8d). Of these species, individuals from 10 shrub species bore ripe fruit beneath black-bear-created gaps, but individuals of 6 shrub species bore ripe fruit beneath closed canopies (Fig 8d).

The best GLMM model found significant and positive effects of forest layers and an interaction between gap size and rPPFDs, and significant and negative effects of the interaction between forest layers and rPPFDs on fruiting or non-fruiting of fleshy-fruited plants (Table 2). Significant effects of life form, canopy disturbance by black bears, gap sizes, rPPFD, and the interaction between canopy disturbances by black bears and rPPFDs were not found for fruiting or non-fruiting of fleshy-fruited plants (Table 2). However, we found negative effects of tree, small tree, and shrub life-forms on the fruiting or non-fruiting of fleshy-fruited plants, so there was a tendency for the fruiting of woody lianas to improve more than that of other life forms (Table 2).

**Table 2:** Results of null and best models selected from all generalized linear mixed models (GLMM) on the effects of canopy disturbances by Japanese black bears, size of black-bear-created gaps, relative photosynthetic photon flux density (rPPFD), forest layers, and life form of any fleshy-fruited plants on the fruiting/non-fruiting of fleshy-fruited plants found beneath Mongolian oaks (*Quercus crispula*).

Model	AIC	Response variable	Explanatory variable	Coefficient	*p*
**Null**	266.8	Fruiting/non-fruiting	1		
**Best (Full)**	239.3	Fruiting/non-fruiting	Life form [Tree]	-5.60	ns
			Life form [Small tree]	-5.31	ns
			Life form [Shrub]	-4.55	ns
			Bear disturbance	-0.52	ns
			Gap size	-0.14	ns
			rPPFD	0.15	ns
			Forest layer	0.45	***
			Bear disturbance * rPPFD	0.08	ns
			Gap size * rPPFD	0.01	*
			Forest layer * rPPFD	-0.02	**

Woody liana was used as a reference life-form category

ns:

*p* ≥ 0.05,

*:*p* < 0.05,

**:*p* < 0.01,

***:*p* < 0.001

## Discussion

### Effect of black bear-mediated canopy disturbance on the fruiting of fleshy-fruited plants

This study generally supported the hypothesis that black-bear-created canopy gaps improve light conditions and facilitate fruiting in fleshy-fruited plants beneath the gaps. Light conditions improved beneath black-bear-disturbed trees with large canopy gaps and the effect of improvement to light conditions declined with descending forest layers (Fig 6, Table 1). Fruiting of fleshy-fruited plants was facilitated by the improvement of light conditions accompanied by the increase in size of black-bear-created gaps (Table 2). Fruiting of plants improved with ascending forest layers (Table 2, Fig 7), and woody lianas and trees in canopy layers had ripe fruit (Fig 8a and 8b). Therefore, our results showed that the canopy layers were key areas of disturbance that had positive effects on light conditions and accelerated fruiting in fleshy-fruited plants, particularly trees and woody lianas with high growth rates and the ability to climb trees. Our findings are generally consistent with previous studies of tropical forests that reported that woody lianas occupy very early canopy gaps formed by tree-fall or large branch-fall and then start rapid growth and reproduction under high-light conditions (see review by [32]).

Although this study focused on light conditions under canopy gaps, other factors affect fruiting by plants beneath black-bear-created gaps. One of these additional factors is increased pollination success. Incidentally, all of the 28 fleshy-fruited plant species observed in this study were insect-pollinated. Generally, the pollination success of a plant varies according to the density of neighboring conspecific and heterospecific plants, which share the same pollinators [33–35]. Plants that occur at low densities receive low pollinator visitation, whereas increases in the density of neighboring plants lead to increases in pollinator visitation [36]. In this study, abundant flowers on the woody lianas and trees concentrated in black-bear-created gaps may also attract many pollinators.

### Indirect effect mediated by a canopy tree between black bears and fleshy-fruited plants

Biological interactions shape biotic communities, alter interspecific competition, and appear to be important drivers of biodiversity [37, 38]. Recent studies on biological interactions have emphasized the importance of indirect effects in shaping biotic communities [39]. An indirect effect occurs when one species can affect a second species only in the presence of a third species [40]. Here, to clearly identify indirect interactions between black bears, fleshy-fruited plants, and canopy trees (i.e., *Q*. *crispula*), we describe the interactions among the three organisms. Interactions between black bears and fleshy-fruited plants are mutualistic because bears carry the seeds of fleshy-fruited plants and the plants provide fruit pulp. Black bears break branches of *Q*. *crispula* trees to feed on acorns; therefore, the interaction between the two species is antagonistic, representing a fundamental consumer—resource linkage in the food web. Because *Q*. *crispula* trees and fleshy-fruited plants use sunlight for photosynthesis, a *Q*. *crispula* canopy tree reduces light availability for fleshy-fruited plants that occur beneath the tree, and thus the interaction between the two organisms involves repression. Therefore, data from this study generated the following hypotheses: (1) the reduction in repression effects due to light is a key component of the indirect effects mediated by *Q*. *crispula* trees between black bears and fleshy-fruited plants, and (2) because black bears disturb the characteristics of a *Q*. *crispula* tree’s canopy but do not affect the overall density of *Q*. *crispula* trees, the indirect effect observed in this study is not a density-mediated indirect interaction, but a trait-mediated indirect interaction [39].

### Japanese black bear as ecosystem engineer

Ecosystem engineers are defined as organisms that directly or indirectly modulate the availability of resources (other than themselves) to other species by causing physical state changes in biotic and abiotic materials [20]. The physical processes that are brought about by ecosystem engineers result in the modification, maintenance, and/or creation of habitats [41]. This concept provides a general framework for understanding interactions between species that are mediated by the direct or indirect effects of certain species on the abiotic environment [42, 43]. Although previous studies have shown that large animals, such as African bush elephants (*Loxodonta africana*), Indian crested porcupines (*Hystrix indica*), and European badgers (*Meles meles*), act as ecosystem engineers [20], no study has focused on the roles of the eight bear species—polar bears (*Ursus maritimus*), giant pandas (*Ailuropoda melanoleuca*), brown bears (*Ursus arctos*), American black bears (*Ursus americanus*), Asiatic black bears (*Ursus thibetanus*), spectacled bears (*Tremarctos ornatus*), sun bears (*Helarctos malayanus*), and sloth bears (*Melursus ursinus*)—found throughout the world.

Our results suggest that Japanese black bears directly increase the availability of light resources to fleshy-fruited plants by causing physical state changes in the canopies of *Q*. *crispula* trees. This leads directly to the facilitation of seed production, which is closely related to plant fitness [44, 45], 2 years after the formation of the gaps. In addition, it suggests that black bears contribute to the provision of food resources for various frugivorous animals by indirectly increasing fruit abundance in forests. Therefore, our data suggest that the Japanese black bear is a potential ecosystem engineer that supplies resources to various organisms. If this hypothesis is supported, black bears may be agents that maintain forest ecosystems and biodiversity in regions with larger black bear populations, such as our study area (3.3–3.9 individuals/km^2^ [21]). To evaluate this hypothesis, we need to determine if interactions with black bears depend on their population density. For example, interactions are largely ineffective in regions with low population densities or without bears.

## Conclusions

We found evidence that fruiting of fleshy-fruited plants in canopy layers is facilitated by the improvement of light conditions caused by small canopy gaps created by black bears, which break the branches of *Q*. *crispula* trees in search of acorns. This indicates that such breaking of branches is an important source of disturbance and acts as a key trigger affecting light conditions and fruit production. Our results thus suggest that the Japanese black bear may act as an ecosystem engineer [20], supplying light resources to fleshy-fruited plants. Further investigation of this notion may contribute to creation of a new field of study on forest gaps, indirect effects among organisms, and ecosystem engineers.
